# Hurdles to horizontal gene transfer: species-specific effects of synonymous variation and plasmid copy number determine antibiotic resistance phenotype

**DOI:** 10.1099/mic.0.001652

**Published:** 2026-01-16

**Authors:** Michael Finnegan, Caroline J. Rose, Jeanne Hamet, Benjamin Prat, Stephanie Bedhomme

**Affiliations:** 1 CEFE, CNRS, Univ Montpellier, EPHE, IRD, Montpellier, France; 2 HydroSciences Montpellier, Univ Montpellier, CNRS, IRD, Montpellier, France

**Keywords:** antibiotic resistance, codon usage preferences, horizontal gene transfer, multi-species approach, synonymous variation

## Abstract

Could codon composition condition the immediate success and the orientation of horizontal gene transfer? Horizontal gene transfer represents a change in the genome of expression of the transferred gene, and experimental evidence has accumulated indicating that the codon composition of a sequence is an important determinant of its compatibility with the translation machinery of the genome in which it is expressed. This suggests that codon composition influences the phenotype and the fitness conferred by a transferred gene and thus the immediate success of the transfer. To directly test this hypothesis, we characterized the resistance conferred by synonymous variants of a gentamicin resistance gene in three bacterial species: *Escherichia coli*, *Acinetobacter baylyi* and *Pseudomonas aeruginosa*. The strongest determinant of the resistance level conferred was the species in which the resistance gene was transferred, very likely because of important differences in the copy number of the plasmid carrying the gene. Significant differences in resistance were also found between synonymous variants within each of the three species, but more importantly, there was a strong interaction between species and variant: variants conferring high resistance in one species confer low resistance in another. However, the similarity in codon usage between the synonymous variants and the host genome only explained part of the phenotypic differences between variants in one species, *P. aeruginosa*. Further investigation of alternative explanations did not reveal common universal mechanisms across our three bacterial species. We conclude that codon composition can be a determinant of post-horizontal gene transfer success. However, there are multiple paths leading from synonymous sequence to phenotype, and sensitivity to these different paths is species-specific.

## Introduction

Horizontal gene transfer (HGT) plays a major role in bacterial evolution. It provides genetic variation, allowing bacterial populations to adapt to changing environments [1]. HGT rates vary amongst bacterial species, and in some cases, these HGT events occur at a greater rate than point mutation, underlining their importance as drivers of evolution [2,3]. Unlike most eukaryotes, which seldom undertake HGT, bacteria can potentially source fully fledged genes and other genetic materials from any other bacterium [4]. However, the horizontal spread of genetic material is not equivalent to simple diffusion, and preferred species-gene(s) associations have been repeatedly found (e.g. [5,6]). Studying the factors shaping and orienting horizontal transfers is key to understanding both bacterial genome evolution and the dynamics of horizontal dissemination of genes, in particular of antibiotic resistance genes. Indeed, resistance to antibiotics arises as an adaptive strategy, both in natural and clinical settings [7,8], and can then spread, notably through the association with mobile genetic elements, to other bacterial populations and to new environmental niches. The strong selection for resistance evolution and dissemination incurred by the clinical use of antibiotics since the 1940s [9] has led to an important burden imposed by antibiotic resistances and is increasingly referred to as a public health crisis [10].

Factors shaping and orienting horizontal transfers have been identified: a number of pre-HGT barriers or limitations explain why genetic material does not move freely (reviewed in [11,13]). The most important of these factors is the requirement for donor and receiver to be in close physical proximity (usually through shared ecology), limited bacterial competency, mobile genetic element host range and resistance to bacteriophage [14]. Once a gene has been transferred, its retention in the receiving genome and its vertical and horizontal propagation depend on the balance between the benefit it provides and the costs associated with its presence in the receiving genome, also called post-HGT barriers or limitations. The benefit is usually in terms of new function, for example, the capacity to use new resources or to resist a drug or a heavy metal [15,16], and often depends on the environment. As for the costs or limitations (reviewed in [17]), they are first linked to the cost of carrying new genetic material, both in terms of replication of this material and in terms of perturbation of the transcriptome of the receiving cells, two phenomena which have been well documented for plasmid-mediated HGT [18,19]. Second, some limitations come from the fact that the product of the introduced gene can interact negatively with other host proteins and perturb the host protein–protein interaction network [12]. Finally, the introduced gene is beneficial only if it is efficiently transcribed and translated by the machinery of the receiving cell.

A key factor mediating the compatibility between a transferred gene and the host cell machinery is thought to be the differences in codon usage preferences (CUPs) between the gene and the new host. CUPs refer to the preferential use of synonymous codons in coding DNA [20]. CUP strongly differs between bacterial species and, to a lesser extent, between regions within a genome [21,22]. Historically, CUP was thought to be the product of mutational bias and drift, with synonymous mutations seen as silent or neutral [23]. However, there are now numerous examples of non-neutral synonymous SNPs (e.g. [24,29]). Additionally, studies employing synonymous variants of a gene have highlighted how varying CUP results in different phenotypes and/or fitness [30,34]. The fitness effects of synonymous mutations and the role of selection in shaping CUP were initially interpreted as being essentially due to translational selection [21]: codon usage frequencies are positively correlated with tRNA gene copy number, such that the codons which are preferentially used are codons for which the copy number of genes encoding the corresponding tRNA is the highest [35,36]. Horizontal transfer of a gene with CUP different from those of the receiving genome thus represents a break in the coevolution between CUP and the translation machinery, as the transferred gene is using many codons for which there are few tRNAs to decode them in the receiving cells. These codons are rare codons in terms of CUP of the receiving genome. Use of rare codons is known to reduce translation efficiency and fidelity, leading to the production of a low quantity of functional protein and the accumulation of truncated and erroneous proteins, potentially toxic for the cell [37,39]. More recently, the fitness effects of synonymous variation have also been linked to non-translation effects, either through its impact on mRNA secondary structure and transcript decay rate or through the presence of nucleotide motifs having a function besides that of encoding an amino acid sequence such as promoters, ribosomal binding sites and restriction sites (reviewed in [40]).

A horizontally transferred gene is likely to have CUP that differs from the receiving genome, and in this context, the fitness effects of synonymous variation could be an important factor determining post-HGT success. Results from comparative genomic approaches support this as they show that HGT occurs more frequently amongst bacteria species with similar tRNA pools [41] and that accessory genes with CUP close to the core genome tend to be retained more often in genomes [42]. On the experimental side, most of the approaches [32,33, 43, 44] find phenotypic differences between synonymous variants, but they only use one model bacterial species, often being *Escherichia coli*, and do not include the across-species dimension of HGT. Additionally, in some of these studies [31,33,45], the gene for which synonymous variants are generated is a fluorescent protein gene. Fluorescent protein genes are not expected to affect fitness and are not commonly transferred horizontally. Although they represent a powerful experimental model to quantify the amount of protein produced, they are not an ideal model to study the role of CUP in shaping and orienting HGT.

We set out to experimentally test the hypothesis that a mismatch between the CUP of a transferred gene and that of the new host would determine the success of an HGT event and orient the transfers between species. To do so, the focal gene, for which synonymous variants were designed, was *aacC1*, a gentamicin resistance gene [48]. *aacC1* produces an aminoglycoside acetyltransferase which provides resistance by transferring an acetyl group to the antibiotic, thus impeding drug fixation on its target, the 30S ribosomal subunit. Besides public health relevance, antibiotic resistance genes represent a convenient model system as the phenotype they confer can be easily determined through classic resistance measures such as MIC [49], and selection pressure is easy to manipulate by varying antibiotic concentrations in the environment. Our biological system was composed of three bacterial species: *Acinetobacter baylyi*, *E. coli* and *Pseudomonas aeruginosa*, with very contrasted CUP (Table S1, available in the online Supplementary Material). The strains we used are each closely related to pathogenic bacteria species or strains [50] which together make up a large proportion of deaths attributed to or associated with antibiotic resistance [10].

We hypothesized that variants which better matched the host’s CUP would provide higher resistance. By introducing our *aacC1* synonymous variant collection into the three chosen bacteria species, we established that they indeed conferred very different levels of resistance to gentamicin within each species. Additionally, the overall mean resistance differed from one species to another, and variant resistances did not rank in identical order across species, revealing an interaction between gene variant and host genome in determining the phenotype. However, in contradiction to our original hypothesis, the average similarity of codon usage between the transferred gene and the host genome was not the main determinant of resistance level for each species-variant combination. The role of local codon usage and internal motifs was then further investigated. Species-specific effects were revealed which highlight both the benefit of our multi-species approach and the difficulty of predicting resistance levels and HGT success, from nucleotide sequences.

## Methods

### Synonymous variant design and synthesis

Synonymous variants of the *aacC1* gene (GenBank: AAB20441.1, *Serratia marcescens*) were designed using the *Optimization Analysis* function of the COUSIN (COdon Usage Similarity INdex) tool [51] and codon usage tables (CUTs) of *E. coli* K12 MG1655 (GenBank: U00096.3), *P. aeruginosa* PAO1 (ATCC 15692, NC_002516.2), *A. baylyi* ADP1 (NC_005966.1) and *Bacillus cereus* ATCC 14579 (GenBank: AP007209.1). *B. cereus* had originally been chosen to include a Gram-positive species. However, the species was discontinued post-cloning after failure to express and maintain the pBBR1 plasmid and *aacC1* gene construct.

In each variant, the first 30 bp of the CDS were conserved to minimize the effects of differences in translation initiation around the translation start site linked to mRNA folding and ribosome access to the ribosome binding site (RBS). To design the variable region, we used the COUSIN analysis tool [51]: for each variant and each amino acid position independently, one of the synonymous codons was assigned in a semi-random manner, based on the frequencies of use in the CUT of one of the host species. Twenty variants were produced for each species (Table S2) using the random guided optimization option in which each synonymous codon is independently assigned based on the frequencies of use in the CUT (Tables S3-S6). An additional five variants were produced in *E. coli* using the reverse frequencies option, which reverses the frequencies of the input CUT. Four variants were produced using the one amino acid – one codon option, with each codon being coded by the most frequently used synonymous codon for each amino acid. This option was used once for each of the four species. An additional variant was produced in *E. coli* using the least frequent codon for each amino acid.

**Fig. 1.**
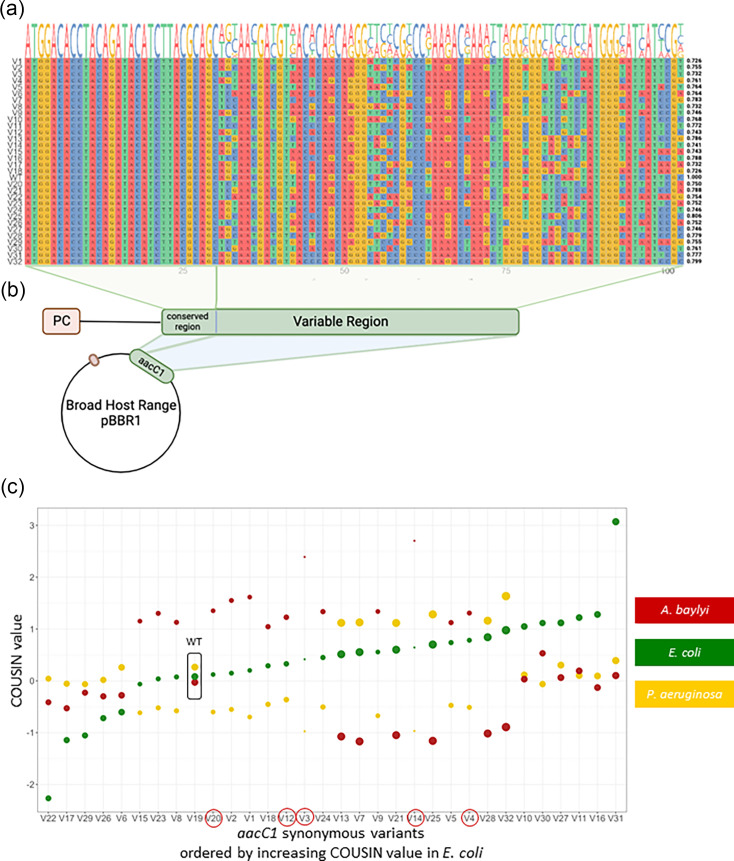
*aacC1* synonymous variant design and constructs. (**a**) First 34 codons of the synonymous variant sequences. The first 10 codons (30 bp) were conserved across variants. The remaining codons were assigned different synonymous codons in a semi-random manner, guided by the codon usage of the bacterial species used. Pairwise identity with wt *aacC1* is shown to the right of each sequence. (**b**) Variant constructs were composed of an integron-derived promoter and 5′ UTR, followed by the *aacC1* gene and cloned into a pBBR1 broad-host-range plasmid. Variant plasmid sequences differed only by the variable region within *aacC1*. (**c**) Codon usage similarity (COUSIN) for the 32 synonymous *aacC1* variants in the three host species used. Variants are ordered by increasing COUSIN values in *E. coli*. GC content at the third base of each codon (GC3) is represented by bubble size. The five variants that could not be transformed into *A. baylyi* are circled in red on the x-axis. Equivalent figures ordered by increasing COUSIN values in *A. baylyi* and *P. aeruginosa* are provided as Fig. S2B and C.

A subset of 31 variants was conserved from the 90 designed variants. Variants were chosen to capture the widest range of COUSIN values in all four species. COUSIN is an index which measures the similarity of codon usage of a focal sequence to a set of reference genes normalized to a null hypothesis of equal usage of synonymous codons. COUSIN and the classical Codon Adaptation Index (CAI [22]) are positively correlated (Fig. S1), but by normalizing to equal usage of synonymous codons, COUSIN allows for direct comparison between genomes [51]. The six variants with the highest COUSIN values in *A. baylyi* and *B. cereus* were chosen (Table S7). The six variants with the highest values were also chosen in *P. aeruginosa*. However, one variant was excluded due to high GC content-related synthesis issues. The five highest values in *E. coli* were retained in addition to four variants produced using the reverse frequencies option. The five one amino acid–one codon option variants were also conserved. The reverse frequencies option was used to provide a better range of mismatch to codon usage in *E. coli* (Figs 1 and S2). For full sequence information on the 31 variants and the wild-type, see Tables S8-S12. Full gene sequences for these 31 variants and the wt variant were synthesized at Twist Bioscience (San Francisco, CA).

### pBBR1 plasmid

The pBBR1 plasmid was ordered from Addgene (pBBR1-MCS-2 plasmid #85168). This broad-host-range plasmid contains a natural plasmid backbone consisting of a pBBR1 *OriV* and pBBR1 *Rep*. The plasmid also contains a kanamycin resistance gene and a multiple cloning site (MCS) [52].

### Variant cloning

The 32 synthesized gene fragments were cloned into the pBBR1 broad host range plasmid using restriction enzyme cloning techniques. They were cloned downstream of a natural integron-derived promoter and 5′ UTR region (Fig. 1b). The resulting plasmid was transformed into DH5α competent cells and selected on kanamycin 50 µg ml^−1^ plates. We Sanger-sequenced successful clones after amplification of a 1,000 bp fragment (containing the *aacC1* variant, 300 bp upstream and ~100 bp downstream) to verify that the variant sequence was the expected one (see Table 1 for primers’ sequence). Because the construction of the variant plasmid did not involve any PCR steps, the likelihood of having mutations in the plasmid backbone, differing between variants, and explaining phenotypic differences is very low.

**Table 1.** Primer sequences

Primer name	Sequence
*E. coli*_rpoD_Fw	TCGAATACCGCCGTGGTTAC
*E. coli*_rpoD_Rv	GCATCTGGCGAGAAATACGG
*P. aeruginosa*_rpoD_Fw	GGGCGAAGAAGGAAATGGTC
*P. aeruginosa*_rpoD_Rv	CAGGTGGCGTAGGTGGAGAA
*A. baylyi*_rpoD_Fw	CGTGGTTTGCAATTCCTTGA
*A. baylyi*_rpoD_Rv	ACCGGAATACGGATGGTACG
pBBR1_*aacC1*_Fw	GTACAGTCTATGCCTCGG
pBBR1_*aacC1*_Rv	GCTGCGTAAGATGTATCTGTAGG

### Bacterial transformation

Plasmids harbouring *aacC1* synonymous variants were transformed into *A. baylyi* (*ADP1*), *E. coli* (K12 MG1655) and *P. aeruginosa* (*PA01*) and plated on selective media. Bacterial clones were isolated, cultured in selective media and archived in glycerol at −70 °C. Species-specific transformation protocols are described in the supplementary methods.

### Bacterial growth curves

Samples were pre-cultured from frozen stocks for 24 h in Lysogeny Broth (LB) without antibiotics. Samples were then transferred to a 96-well microplate containing serially diluted concentrations of gentamicin and an LB control. Growth curves were produced by incubating samples at 30 °C (*A. baylyi*) or 37 °C (*P. aeruginosa*, *E. coli*) for 24 h (48 h for *A. baylyi*) in a Spark Tecan spectrophotometer. A humidity cassette was employed to prevent evaporation. Growth was recorded by measuring absorbance at 600 nm at T0 and approximately every 30 min for 24 h or 48 h. During each 30 min cycle, an absorbance measure was recorded, followed by a 22 min wait period, followed by a 5 min orbital shaking period (108 r.p.m., 2.5 mm amplitude). This was then followed by a 2 min settlement period. Growth curves were performed in triplicate, with each measurement performed on a different growth plate assay, on a different day, using separate streaks of the frozen stock culture.

### Growth curve analysis

Growth curve data was analysed using the R package gcplyr [53]. The growth metrics maximum growth rate, carrying capacity (Max OD), lag time and area under the curve (AUC) were calculated from individual growth curves for each replicate, for each gentamicin concentration, for each variant and for each species. Individual growth metrics are available in Tables S13 to S15.

### Resistance levels (IC50_AUC_ and MIC calculations)

Half maximal inhibitory concentration (IC50_AUC_) is defined here as the concentration of gentamicin that resulted in a 50% reduction in AUC compared to growth in the absence of antibiotics. IC50_AUC_ was calculated using a four-parameter log-logistic fit of the drm() function in R (drc package).

MIC was determined from growth curve data. We defined MIC as the lowest gentamicin concentration at which final OD was <0.1.

### Plasmid copy number determination

Plasmid copy number (PCN) was quantified by qPCR of a 100 bp sequence in the 5′ UTR of the *aacC1* gene and of a 100 bp sequence in the *RpoD* gene (see primers’ sequences in Table 1). *RpoD* is present in a single copy on the chromosome of each species. PCN was calculated with the following formula:

(qPCR_efficiency_reference^Ct_reference_gene)/(qPCR_efficiency_target^Ct_target_gene)*(reference_gene_size_bp/target_gene_size_bp)

qPCR was conducted in triplicate on three *aacC1* variants (V2, V11 and V19) in each of the three species. The qPCR conditions were as follows: LightCycler^®^ 480 SYBR Green I Master (Roche), 45 cycles on LC480 (Roche) – 94 °C for 10 s, followed by 66 °C (*rpoD* primers) or 59 °C (pBBR1 aacC1 primers) for 10 s, followed by 72 °C for 10 s.

### Codon usage preferences metric calculations

COUSIN and CAI were calculated using the COUSIN tool (https://cousin.ird.fr/calculation.php), by inputting the CUT for each of the three species and the 32 variant CDSs. CAI here is calculated on the frequencies of synonymous codons across the genome and not in a subset of highly expressed genes. COUSIN was calculated as described in Bourret *et al*. [51].

tAI (tRNA Adaptation Index) was calculated using the tai R package (https://github.com/mariodosreis/tai), by inputting tRNA copy number for each species and the variant CDSs.

 |Δ(GC3)| was calculated as the absolute value of the difference in GC3 between a variant sequence and the host genome. GC3 for each variant was obtained using the COUSIN tool calculation. GC3 for each species was taken from CoCoPuts described in Athey *et al*. [54].

### Local codon usage metrics

The *aacC1* gene was split into 30 bp sliding windows (steps every 15 bp). CAI, COUSIN, tAI and |Δ(GC3)| were calculated for these 36 windows as above.

Protein structural domains of the *aacC1* gene CDS were extracted from https://www.ebi.ac.uk/pdbe/entry/pdb/1bo4/analysis (EMBL-EBI Protein Data Bank in Europe, 1bo4 [55]).

Bottleneck position and strength were calculated similarly to Navon and Pilpel [56]. Bottleneck position is defined here as the position of the window with the lowest tAI value. Bottleneck strength is defined here as the tAI of the bottleneck relative to the mean tAI of the 36 sliding windows (bottleneck tAI/mean tAI).

Rare codon chains were defined as the number of chains of 1, 2, 3, 4 or 5 rare codons, calculated using the str_count() in R. We define rare codons for each species as codons that are underrepresented in the host CUT. More specifically, we defined rare codons as those with less than half their expected frequency under the null hypothesis of equal frequency within a synonymous codon family (e.g. for a codon within a four-synonymous-codon family, a rare codon is a codon with a frequency <0.125).

### Shine–Dalgarno-like sequences

Shine–Dalgarno (SD)-like sequences were defined as hexamers with high affinity to the conserved anti-SD sequence. This was done by calculating the free binding energy between CACCUCCU and all possible hexamers within the *aacC1* gene variants using the RNAup function of the ViennaRNA 2.0 Package [57]. We counted the number of hexamers in each synonymous variant with a binding free energy < −4 kcal mol^−1^, which is the definition of a weak SD-like in Hockenberry *et al*. [58]. A strong SD-like sequence is defined as < −7 kcal mol^−1^.

### mRNA secondary structure

mRNA folding energy was calculated using the Vienna RNA 2.0 function RNAfold, by inputting the −30 : 60 bp section of each variant mRNA sequence (1 : 3=ATG of the CDS).

### Statistical tests

All one-way and two-way ANOVA tests and Pearson correlation tests were performed in R using the functions aov() and cor.test() (stats package), respectively. *P*-values were corrected using the Benjamini–Hochberg (BH) critical value (i/m)Q in R, where i=individual *P*-value’s rank, m is the total number of tests and Q is the false discovery rate (FDR) [59].

## Results

### Design of synonymous gene variants to match and mismatch the CUPs of three bacterial host species

We designed synonymous variants of the *aacC1* gene by varying codon positions 20 to 183 (Fig. 1a, 31–549 bp). We specially designed this variable region to match the codon usage of each of the bacterial species (Figs 1c and S2).

In order to minimize the effects of differences in translation initiation around the translation start site due to mRNA folding, we conserved the first 30 bp of the *aacC1* sequence. Recent studies [31,38] suggest that synonymous variation at the 5′ end of the CDS is responsible for the lion’s share of phenotypic differences between synonymous variants, through the effect of the nucleotide sequence on mRNA folding energy and consequently on ribosome accessibility to the ribosomal binding site. Thus, we choose to conserve this region in order to untangle the effects of CUPs on translation initiation and on translation efficiency and accuracy. The stop codon was also identical for all variants.

The 31 synonymous variants (and the wild-type sequence) of the *accC1* gene covered a wide range of codon usage match within each species and presented important shifts in codon usage match from one species to another (Figs 1c and S2). The 32 variants also differed in GC content (Fig. 1c) and had pairwise identities to the wt ranging from 0.726 to 0.806 (median=0.755) (Fig. 1a). The pairwise identity between variants ranged from 0.621 to 0.940 (median=0.759) (Fig. S3). The third codon GC content (GC3) of the variants ranged from 5.98% to 98.37% (median=44.57%, wt=59.78%).

The 32 plasmid versions, each carrying one synonymous variant, were successfully introduced in *E. coli* and *P. aeruginosa* by electroporation. Twenty-seven of the variants were introduced into *A. baylyi* by natural transformation. The remaining five variants could not be transformed (Fig. 1c), and we did not find any obvious reason for this. We noted that these non-transformed variants show high COUSIN values in *A. baylyi* compared to the 27 transformed variants (mean COUSIN value of the 5 variants=1.80, mean COUSIN of the transformed 27 variants=0.24), but three of them had COUSIN values similar to those of transformable variants. The 5 variants also have much lower GC3 content (mean GC3 of the 5 variants=19.13%, mean GC3 of the transformed 27 variants=50.58%), but again, the majority of them are in the lower range of GC content of the transformable variants.

### Synonymous variation modulates resistance levels in three different bacterial hosts

We chose to measure the resistance phenotype conferred by the variant to the three species as our main aim was to determine the effects of CUPs on resistance levels following HGT. Specifically, we were interested in the changes in phenotype of a variant when transferred into a new host, with different transcriptional and translational machinery. RNA and protein quantification would provide additional interesting information but would not fully resolve the mechanistic link between genotype (synonymous variant) and phenotype (resistance level). Indeed, RNA-Seq results combine transcription levels and mRNA decay rates [60], both of which are potentially influenced by the nucleotide composition of the synonymous variant. Additionally, protein quantities are not fully indicative of quantities of functional protein, and it is known that codon usage can influence both translation errors (potentially producing erroneous, non-functional proteins) and protein folding [34,61]. Additionally, codon usage could have an effect through differential mRNA toxicity depending on the nucleotide sequence [33].

Resistance levels were determined by growing all *aacC1* synonymous variant * species combinations across a range of gentamicin concentrations. We used two variables to represent resistance to gentamicin in our variants, MIC and IC50_AUC_. The AUC metric was chosen as it integrates information about lag time, growth rate and carrying capacity [62]. AUC, growth rate and lag time measures for each variant in each gentamicin concentration are available in the supplementary materials (Figs S4–S6).

**Fig. 2.**
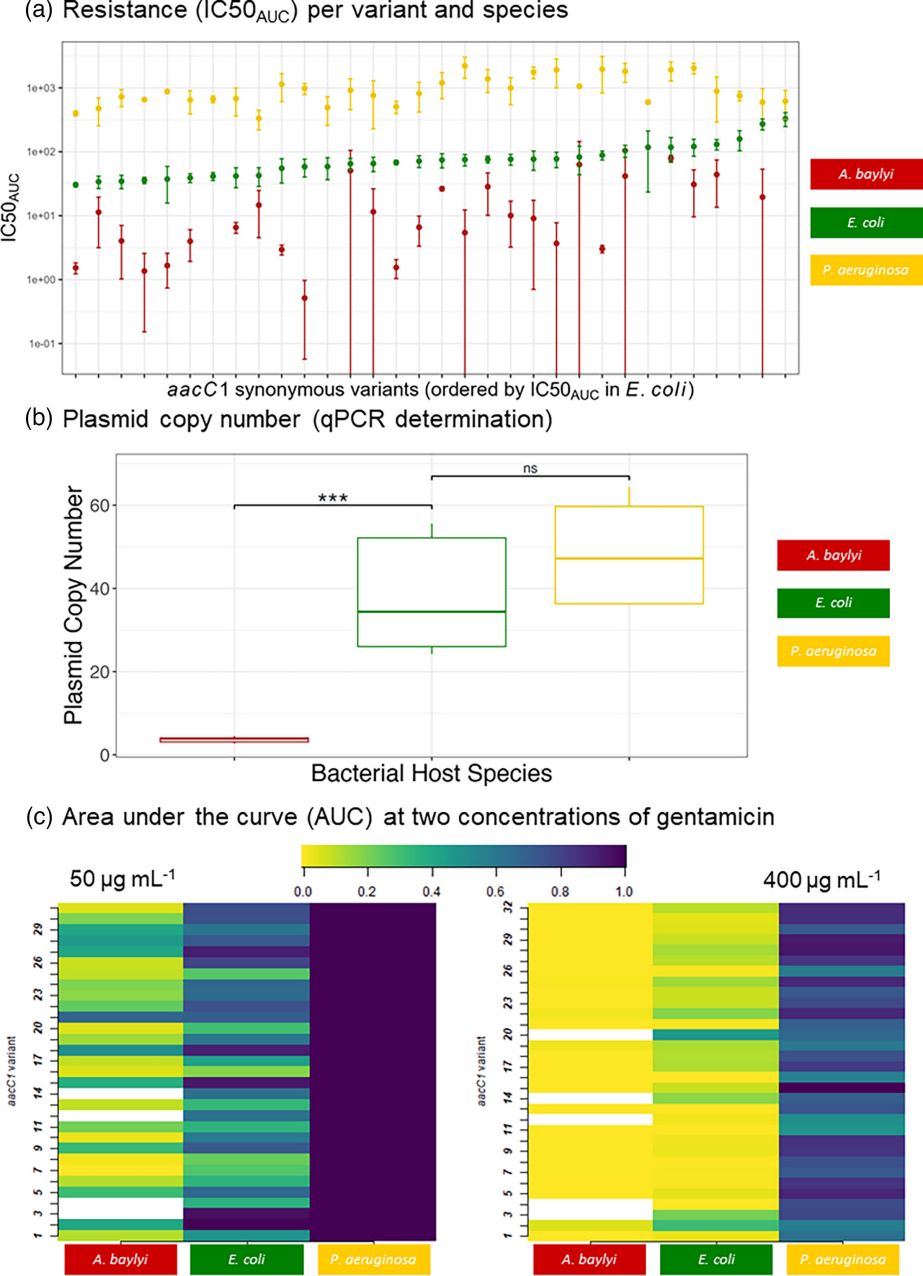
Synonymous variants confer different resistance levels. (**a**) Resistance levels are represented by IC50_AUC_, i.e. the concentration of gentamicin required to reduce the AUC by 50% compared to growth in the absence of gentamicin. Synonymous variants are ordered on the x-axis by increasing IC50_AUC_ in *E. coli*. (**b**) PCN, quantified by qPCR, in each host variant. Significant differences in PCN are indicated by ***, and non-significant differences are indicated by ns. (**c**) AUC of the three bacteria species carrying the *aacC1* variants in 50 µg ml^−1^ and 400 µg ml^−1^ of gentamicin. AUC is represented as relative to AUC in 0 µg ml^−1^ gentamicin. Some variant*species combinations were not tested at each of the concentrations. The AUC of these combinations was set to 0 when the variant*combination did not grow at a lower gentamicin concentration and to 1 when the variant*combination had, in the presence of a higher concentration of gentamicin, an AUC equivalent to the one in the absence of gentamicin. White boxes indicate the five variants that could not be transformed in *A. baylyi*.

We found a strong statistically significant interaction between species and variants for IC50_AUC_ (two-way ANOVA on data for variants that could be transformed in the three species, F_52,161_=3.94, *P*<0.0001). Species and variants had significant effects (species, F_2,161_=375.15, *P*<0.0001; variant, F_26,161_=4.32, *P*<0.0001). Mean IC50_AUC_ ranged from 18 µg ml^−1^ gentamicin in *A. baylyi* to 1,097 µg ml^−1^ in *P. aeruginosa* (Fig. 2a). The variant effect on IC50_AUC_ was also significant within each species (Table S16): the modulatory capacity of synonymous variation on resistance levels held across all three bacterial species, as illustrated in Fig. 2(a), with IC50_AUC_ differing by 154.4-, 10.78- and 6.65-fold in *A. baylyi*, *E. coli* and *P. aeruginosa*, respectively. MIC values ranged in *P. aeruginosa* from 800 µg ml^−1^ to >25,600 µg ml^−1^, whilst they ranged from 25 µg ml^−1^ to >25,600 µg ml^−1^ and 6.25 µg ml^−1^ to >400 µg ml^−1^ in *E. coli* and *A. baylyi*, respectively (Fig. S7).

Given the strong species effect (Fig. 2a), we sought to determine if differences in resistance levels were due, in part, to differences in PCN. We quantified PCN, the number of plasmid copies, in each bacterial species by qPCR and observed significant differences (one-way ANOVA, F_2,6_=13.16, *P*=0.0064, Table S17) in copy number between species (PCN: *A. baylyi* 3.67+/−0.65, * E. coli* 38.00+/−12.60, *P. aeruginosa* 48.72+/−11.37). Notably, PCN ranked in the same order as the average resistance level in each species (Fig. 2a, c). To assess whether PCN explains interspecies variation in resistance level, we compared two linear mixed-effects models: one with species as a random effect and variant as a fixed effect, and a second model additionally including PCN as a fixed effect. Including PCN did not improve the model fit (ΔAIC=–0.1, χ²=2.13, *P*=0.144). Notably, the marginal R² increased from 0.069 to 0.250 with the inclusion of PCN, indicating that PCN accounts for an additional ~18.1% of the variance in IC50 explained by fixed effects. However, the species-level random effect variance decreased only slightly (–1.7%), and the effect of PCN was not statistically significant (t=1.02). These results suggest that while PCN contributes meaningfully to variation in resistance, it does not explain the majority of the species effect, which likely reflects other unmeasured species-specific traits. 

The combination of the species and synonymous variant effects in determining the resistance level is very likely to affect the immediate success of HGT, as illustrated in Fig. 2(b): the AUC at a given gentamicin concentration differs strongly depending on the receiving species and the received synonymous variant. In the 50 µg ml^−1^ environment, all of the *P. aeruginosa* grow as well as in the absence of antibiotics, but when the variants are transferred into *E. coli*, there is a large range of AUC levels (from 0.1 to 1), showing that some variants permit growth and others have a clear disadvantage post-HGT. This effect is even more pronounced in *A. baylyi*, suggesting that many of the variants, if transferred into a 50 µg ml^−1^ environment, would not succeed post-HGT and be selected against. At 400 µg ml^−1^, *P. aeruginosa* variants grow at different rates (AUC range from 10.48 to 23.06), influencing their post-HGT success in the environment. Inversely, almost all of the variants, when transferred into *E. coli* and *A. baylyi*, show very little growth, preventing post-HGT success in 400 µg ml^−1^ gentamicin. The significant ‘variant’ effect actually holds in most individual gentamicin concentrations for AUC, lag maximum growth rate and OD max (Table S18).

### Similarity with host CUPs is not a strong determinant of variant resistance phenotype

The CAI was first described by Sharp and Li [22] and linked the biassed use of synonymous codons in highly expressed genes to their gene expression, putting forward the hypothesis that codon usage bias (CUB) is correlated with expression and thus potentially under selection. Since then, a multitude of codon usage indexes have been developed to piece apart the relationship between CUB and phenotype, through their impact on transcription, translation, mRNA stability and co-translational folding (reviewed in [41]).

We sought to determine if the observed differences in resistance phenotype could be explained by the effect of CUP on translation efficiency and fidelity. To do so, we used different codon usage indexes: CAI [22], COUSIN [51] and also tAI which measures the match between the CUP of a sequence and the tRNA gene pool of the host genome [63]. The last index used was |Δ(GC3)|, measuring the absolute value of the difference in GC3 between a sequence and the host genome.

**Fig. 3.**
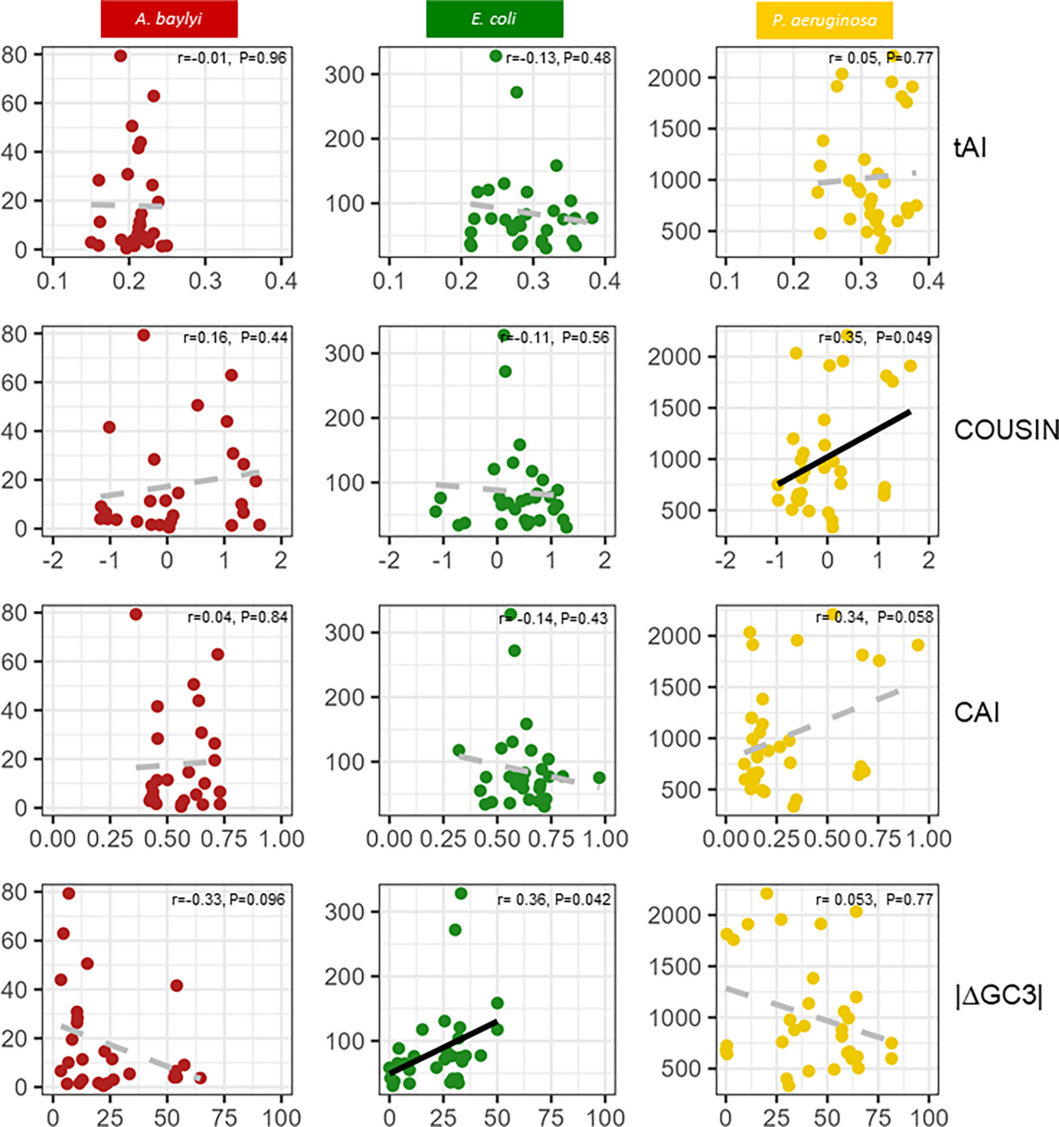
CUPs and resistance. Pearson correlation between resistance (IC50_AUC_) and tAI (first line), COUSIN (second line), CAI (third line) and |Δ(GC3)| (fourth line) for *A. baylyi* (first column), *E. coli* (second column) and *P. aeruginosa* (third column). Significant correlations (*P*<0.05) are represented by a black regression line. Non-significant Pearson correlations are represented by a grey dotted regression line.

A significant positive correlation was found between COUSIN and resistance levels in *P. aeruginosa* (r=0.35, *P*=0.049) but not in the other two species (Fig. 3b). No significant correlation existed between CAI or tAI and resistance level in either of the species (Fig. 3). We also find a significant positive relationship between |Δ(GC3)| and resistance in *E. coli* (r=0.36, *P*=0.042). We further explored correlations between codon usage indices and AUC within individual gentamicin concentrations (Table S19). While IC50_AUC_ measures allow us to determine resistance levels for each variant, individual concentrations can be regarded as unique environments in which a variant may be horizontally transferred. In agreement with the inter-concentration effect, we observed significant correlations between AUC and COUSIN in *P. aeruginosa* at high gentamicin concentrations and between AUC and |Δ(GC3)| in *E. coli* at intermediate concentrations. Additionally, we observed a number of significant correlations in individual gentamicin concentrations that were masked in the IC50_AUC_ results, such as positive correlations between AUC and CAI in * P. aeruginosa* and between AUC and |Δ(GC3)| in *A. baylyi* and a negative correlation between AUC and |Δ(GC3)| in *P. aeruginosa* for high gentamicin concentrations.

The results presented here provide some insight into the mechanisms determining the modulatory effect of synonymous variation on resistance levels and bacterial growth and fitness in the presence of antibiotics. However, codon usage similarity averaged over the gene only partially explains the phenotypic differences between synonymous variants in two out of the three species. This prompted us to explore the relationship between resistance and local codon usage.

### Localized sequences within the *aacC1* gene partly modulate resistance phenotype

Overall similarity in CUP to the host genome could not fully explain the large variation of resistance levels amongst the *aacC1* variants. But it is possible that the effects are due to the codon usage of key positions or segments of the variants and that these effects are not captured when using CUP indices calculated over the whole variant sequence. To try and reveal these potential local codon usage effects, we calculated CAI, COUSIN, tAI and |Δ(GC3)| values for 30 bp sliding windows across the *aacC1* gene, with 15 bp steps. This window size was chosen as it represents the ribosome footprint [64]. The ribosome footprint, or rather the 10 codons (30 bp sequence) covered by an individual ribosome, is predicted to influence translation elongation rates and translational pauses. Translational elongation rates and pausing in turn influence protein folding and possibly translation abortion rates [65], modulating protein levels and protein quality and thus potentially protein function, phenotype and fitness.

**Fig. 4.**
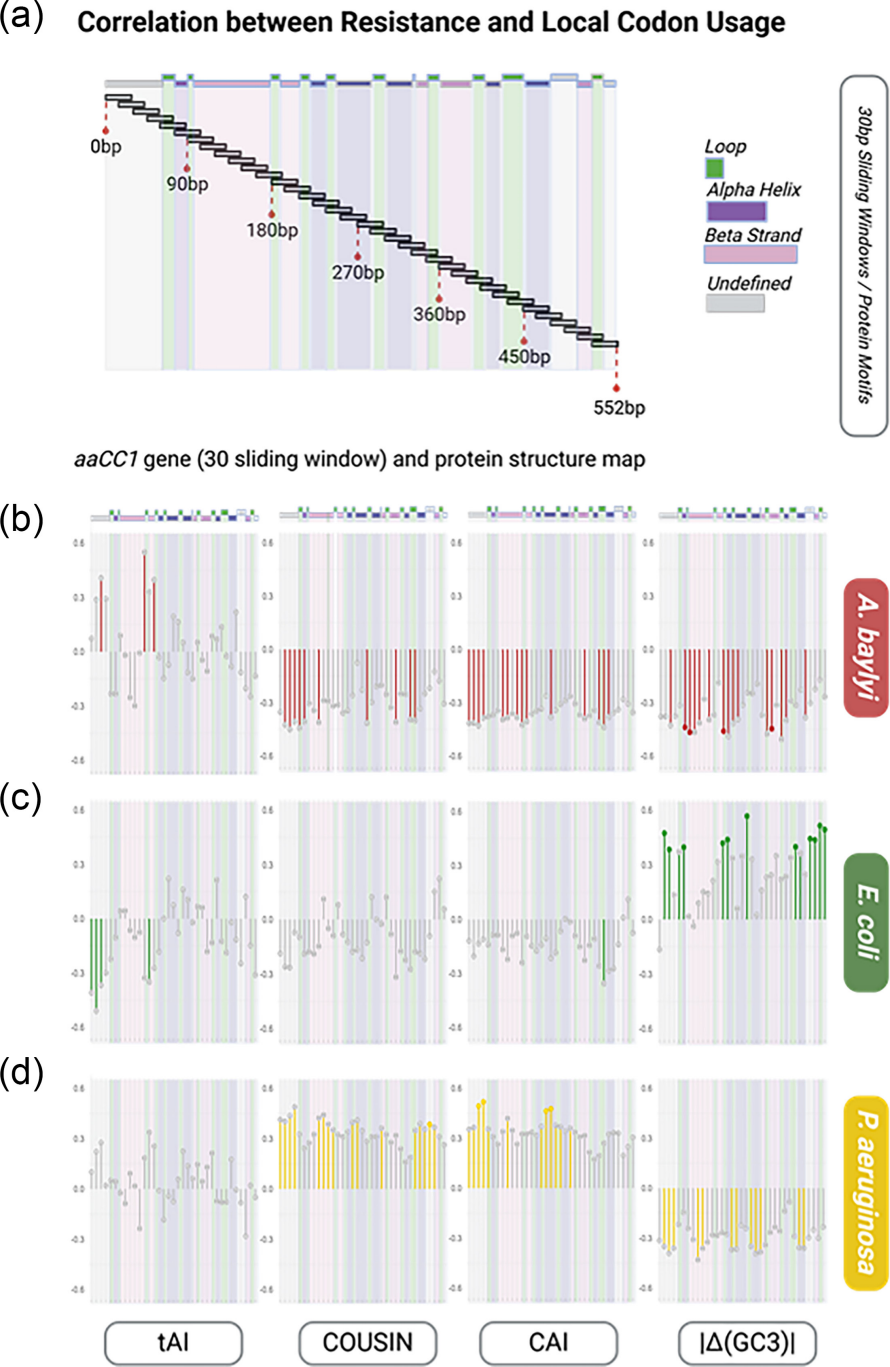
Local codon usage and resistance with BH FDR corrections (a). Protein structural elements, i.e. alpha helices, beta sheets and loops, are represented in purple, pink and green, respectively. The positions of these elements within the CDS are displayed above the sliding window. Undefined elements are represented in grey. (b–d) Pearson correlation between resistance (IC50_AUC_) and tAI, COUSIN, CAI and |Δ(GC3)| (from left to right) at 30 bp-sized sliding windows (sliding every 15 bp) of the *aacC1* gene, represented by r on the y-axis. Lollipop graph icons represent the r value for each of the 35 sliding windows, ordered from 0 to 30 bp to 525–552 bp. Window 0–30 bp is common to all *aacC1* variant sequences and is thus excluded from correlation tests and left blank. Non-significant correlations (*P*-value<0.05) are represented in grey. Windows for which the *P*-value is <0.05 are represented by a coloured lollipop stalk. Windows for which the correlation remains significant after BH FDR (*P*=0.1) are represented by a coloured lollipop head.

We then looked for correlations between variant resistance levels (IC50_AUC_) and the codon index values of individual 30 bp windows. Results of Pearson correlation analyses by sliding window are available in Table S20. Unsurprisingly, we captured significant correlations in sliding windows between COUSIN and resistance for *P. aeruginosa* (16 out of 35 sliding windows, Fig. 4d) and between |Δ(GC3)| and resistance in *E. coli* (13 out of 35 sliding windows*,* Fig. 4c), consistent with the results obtained with the codon usage index averaged across the whole gene. In *P. aeruginosa,* the significant positive correlations with COUSIN values were localized in the regions 15–90 bp, 135–210 bp and 450–525 bp (Fig. 4d). In *E. coli*, the significant negative correlation with |Δ(GC3)| was distributed across the sequence (Fig. 4c).

COUSIN shows significant negative correlations with resistance in *A. baylyi* (10 out of 35 sliding windows, Fig. 4b). No such significant correlation was observed in *E. coli* (Fig. 4c). In *A. baylyi*, these significant negative correlations were localized at the beginning of the sequence 45–105 bp and at 420–465 bp (Fig. 4b), partially similar to the location in *P. aeruginosa*.

For |Δ(GC3)| content, we observed significant negative correlations with resistance in *A. baylyi* (15 out of 35 sliding windows, Fig. 4b). In *P. aeruginosa*, we observed significant negative correlations (12 out of 35 sliding windows, Fig. 4d). In *A. baylyi*, these resistance–|Δ(GC3)| negative correlations are localized from 30 to 120 bp, from 270 to 315 bp and from 420 to 480 bp (Fig. 4b). In *P. aeruginosa*, the positive correlations are from 135 to 195 bp, from 255 to 375 bp and from 450 to 540 bp (Fig. 4d).

For CAI, significant negative correlations with resistance were observed in *A. baylyi* (14 out of 35 sliding windows), and significant positive correlations were observed in *P. aeruginosa* (12 out of 35 sliding windows), mirroring the results for COUSIN (Fig. 4c). No significant correlation was observed in *E. coli*. In *A. baylyi*, this resistance vs CAI negative correlations are localized from 15 to 105 bp, 180–225 bp and 420 to 465 bp. In *P. aeruginosa*, the positive correlation was from 15 to 105 bp and from 240 to 360 bp (Fig. 4c).

For tAI, there was no significant correlation in *P. aeruginosa* (Fig. 4d). In *A. baylyi*, three sliding windows (45–75 bp, 180–210 bp and 210–240 bp) correlated positively with resistance (Fig. 4b). In *E. coli*, resistance correlated negatively with tAI in four windows (15–75 bp and 195–225 bp) (Fig. 4c).

It has to be noted that the majority of correlations are not significant anymore when FDR correction is applied (Fig. 4). The FDR chosen for this experiment was 0.1.

The local effects detected are thus not located on the same segment of the gene in the three species nor across the different indices, and they do not seem to correspond to secondary structure of the protein (Fig. 4a). This was further confirmed by the absence of correlation between the codon usage indices and the resistance level for the concatenation of the segments belonging to each of the four secondary structure classes (see Table S21).

### Translational bottlenecks do not explain differences in resistance levels between synonymous variants

**Fig. 5.**
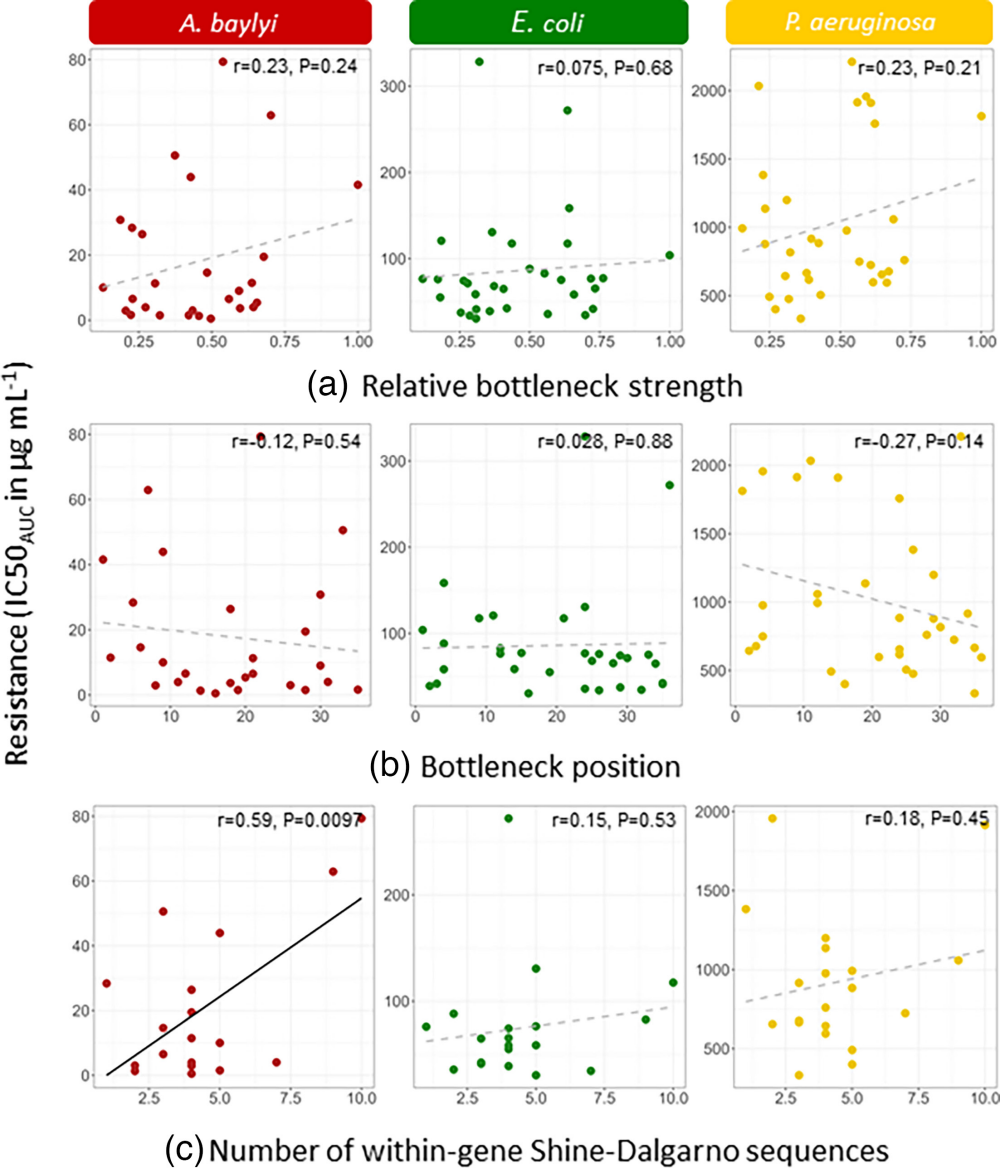
Transcriptional and translational motifs and resistance. (**a**) Correlation between resistance (IC50_AUC_) and relative bottleneck strength. Relative bottleneck strength is the minimum tAI value divided by the mean tAI value of the 36 sliding windows. (**b**) Correlation between resistance (IC50_AUC_) and bottleneck position, expressed as the position (from 1 to 36) of the sliding window with the lowest tAI. (**c**) Correlation between resistance (IC50_AUC_) and the number of hexamers within the *aacC1* variant with < −7 kcal mol^−1^ binding free energy (strong anti-SD-like sequence). Significant Pearson correlations (*P*<0.05) are represented by a black regression line. Non-significant Pearson correlations are represented by a grey dotted regression line, and corresponding *r-* and *P-*values are given in the upper left corner of each graph.

Another way through which local codon usage could affect the level of resistance is the presence, length and position of translational bottlenecks. To test this hypothesis, we have used two ways of defining and quantifying translational bottlenecks. First, following Navon and Pilpel [56], we identified translational bottlenecks as the 30 bp window with the lowest tAI. However, we found no significant correlation between bottleneck position or strength and resistance levels in the three host species (Fig. 5a, b).

Second, we tested for a correlation between resistance and the number of rare codon chains. We hypothesized that a high number of rare codons and high frequencies of rare codon chains would produce translation bottlenecks slowing down translation, increasing translation errors and potentially leading to ribosome abortions which can occur during prolonged translational pausing. We tested for correlations between the number of rare codons and resistance levels and also between the number of chains of two, three, four and five rare codons and resistance levels. We did not find any significant relationship in any of the species (Table S22).

### Internal SD-like motifs and secondary mRNA structure do not explain differences in resistance levels between synonymous variants

 The SD sequence, or bacterial RBS, is complementary to the 3′ end of the 16S rRNA of the ribosome and controls translation initiation. Within gene SD or SD-like sequences are expected to have deleterious effects because they can cause within-gene translation initiation and generate ribosome conflicts. Their presence has been shown to impose selective constraints on the surrounding sequences [58]. It is possible that our semi-random design of synonymous variants may have introduced within-gene SD-like sequences, and we hypothesized that there would be a negative relationship between the frequency of within-gene SD-like sequences and resistance. To test this, we looked for a potential relationship between the number of hexamers with a strong affinity to the anti-SD sequence and the resistance level. We found no significant relationship in either *E. coli* or * P. aeruginosa* and a significant positive correlation between the number of anti-SD-like hexamers (< −4 kcal mol^−1^ free binding energy) [58] and resistance in *A. baylyi* (Fig. 5c, Table S23). This significant positive relationship remained when tested in strong anti-SD-like sequences (< −7 kcal mol^−1^ binding free energy, Table S23). This result, being contrary to our prediction, led us to look for a potential explanation: the *aacC1* wild-type sequence used, initially found in *S. marcescens*, shares almost 100% sequence alignment with a shorter, *P. aeruginosa*-derived *aacC1* variant, bar the first 69 bp. Thus, the first 87 bp (including the 18 bp AU1 epitope following ATG of our variants) are not required for gentamicin acetyltransferase activity, and the shorter protein variant could potentially confer resistance more efficiently than our *aacC1* variant. We thus split the *accC1* gene into two segments and correlated resistance and anti-SD-like sequence frequency separately for the beginning 87 bp segment and the ending 465 bp segment (required for enzymatic activity). We hypothesized that the positive correlation between resistance and anti-SD-like frequency was mainly due to the 87 bp fragment. Surprisingly, the significant positive correlation we observed in *A. baylyi* in the full sequence was lost in the 87 bp segment but maintained in the remaining 465 bp segment (Table S24).

Finally, the *aacC1* variants were identical for the first 30 bp to limit differences between variants in the 5′ end secondary structure of the mRNA and the associated effects on translation initiation. However, previous studies (e.g. [31]) identified an effect of mRNA structure of a longer mRNA stretch. We tested whether this was also the case in our synonymous variants by looking at the correlation between mRNA folding energy in the 90 bp around the start codon (−30 : 60 bp) and resistance. We found no significant correlation in any of the three species (Table S24).

## Discussion

We have shown that synonymous variants of a gentamicin resistance gene confer strongly different resistance levels in three distant bacterial species: *A. baylyi*, *E. coli* and *P. aeruginosa*. Our resistance measures show a strong species effect, explained in part by differences in PCN. Additionally, there is an important variant*species interaction, with the variants conferring the highest resistance being different from one species to another. This species-specific effect of synonymous variation points to codon composition as a factor influencing immediate post-HGT success and orienting HGT. However, the average similarity in codon usage between a synonymous variant and the genome of the recipient species only explains a limited part of the variation in resistance levels in one of the species, *P. aeruginosa*.

The strongest determinant of the resistance conferred by a synonymous variant of the *aacC1* gene was the species in which it was introduced. The copy number of the broad-host-range plasmid pBBR1, used as a vector for the synonymous variants, strongly differs between the three host species used. The three species rank in the same order for PCN and average gentamicin resistance level conferred, and we evaluated that PCN is responsible for about 18% of the between-species variance in resistance. The link between species-specific PCN and resistance is likely to be through a resistance gene dosage effect. Different mechanisms of PCN regulation have been described [66,67], but they all involve only plasmid sequences coding for negative regulation mechanisms, such as antisense RNA. Previous studies have linked chromosomal point mutations and PCN in *E. coli* [68,72], but the interspecific variation in PCN is not broadly documented, and the mechanisms behind it are not known. Interestingly, though, Pena-Gonzalez *et al*. [73] report copy number variation for two plasmids across *B. cereus* and *Bacillus anthracis* strains with no phylogenetic signal in the PCN variation but a positive correlation between the copy number of the two plasmids, suggesting a host-driven copy number control common for the two plasmids. Very recently, Alonso-del Valle *et al*. [74] documented copy number differences for the same antibiotic resistance plasmid between species (*E. coli* and three *Klebsiella* species) and between clinically relevant strains within species. Additionally, they report a positive correlation between PCN and the level of resistance conferred. All together, these results strongly suggest that PCN variation across strains and species has implications for immediate HGT success through gene dosage, as genes transferred horizontally on plasmids will produce different phenotypes depending on the PCN in the new host.

However, in our experimental system, PCN explains only 18% of the interspecies variance in resistance, meaning that there are other species-specific traits involved in resistance level determinism. Different transcription levels from the same promoter across the three species are likely to be one of these traits. By transferring a library of regulatory elements (RE) into three bacteria species (*Bacillus subtilis*, *E. coli* and *P. aeruginosa*), Gomes *et al*. [75] show a generally lower promoter activity for the same RE in GC-poor species. Interestingly, out of the three species they use, two are in common with our experimental system, and the third (*Bacillus subtilis*) has a GC content similar to the one of our third species, *A. baylyi*. Their results suggest that the expression of *aacC1* would be higher in *P. aeruginosa* than in *E. coli* and higher in *E. coli* than in *A. baylyi*, an effect coherent with the species-specific resistance levels we obtained. The modelling approach in Alonso-del Valle *et al*. [74] established that in their system, the level of resistance conferred to a strain was a strong determinant of the resistance gene-strain association and of the community composition. In the longer term, PCN is also known to influence the probability of segregational loss, and so plasmid stability, as well as the evolutionary dynamics of the genes carried [76]. Strain- and species-specific PCN and expression level are thus very likely to be important factors in determining the post-HGT success and evolution and in orienting HGT over the long term. Understanding the host PCN regulation mechanisms, as well as host-specific expression levels, is thus important for a better understanding and prediction of antibiotic resistance propagation.

The differences in resistance conferred by our synonymous variants within each of the three species used illustrate the potential of synonymous variation to modulate the phenotype, likely through the expression level of genes and the functionality of the proteins they encode. Our results are in line with previous studies, which have shown that synonymous variations impact mRNA and protein quantities [32,77], mRNA toxicity levels [33], protein folding [34], enzymatic activity levels or protein functionality [30,77, 78] and lead to differences in growth rates or fitness [33,44, 77, 78]. Taken together, they show that synonymous variation affects cell function on multiple levels from mRNA levels to protein levels and protein folding, producing measurable fitness effects. However, all previous studies have been conducted within one species, *E. coli* for a majority of them, and do not provide information of the species specificities of these effects and do not test directly the implications for HGT. Our approach, by introducing the same collection of synonymous variants in three distant species with contrasted codon usage, allowed both to tease apart species-specific effects of synonymous variation and to mimic the horizontal transfer of a same variant across species.

Phenotypic characterization of the *aacC1* variants actually showed that the relative levels of resistance conferred by each synonymous variant were not conserved across species. These species-specific phenotypic effects indicate differing compatibility between the transferred variant genes and the receiver bacterial genomes, with individual variants being more compatible in one genome and less compatible in another. This compatibility has clear implications for HGT outcomes. For example, a variant which confers a certain level of resistance in one species and provides a fitness advantage in the presence of antibiotics may potentially be transferred into other species in the bacterial community. However, if the variant is incompatible or less compatible with the new host species, the resistance phenotype will not manifest, and no fitness advantage will be conferred, preventing the retention of the transferred gene and its further propagation.

A potential mechanism determining the compatibility of a synonymous variant to a new host species is the similarity in codon usage between the variant and the host genome. Hence, we originally hypothesized that variants would differ in translation efficiency and accuracy, resulting in resistance levels that correlated with codon usage similarity indices. Interestingly, the resistance conferred by a variant in a species did not generally correlate with average codon usage similarity indices. We did, however, find a significant correlation between resistance and COUSIN in *P. aeruginosa* and between resistance and |Δ(GC3)| content in *E. coli* (Fig. 3). We further reasoned that the synonymous variants differed by a large number of positions and that the global effect might not be due to the sum of small individual effects but due to the effects of a limited number of these positions. Otherwise said, codon usage might have localized effects that become diluted and thus masked when considering codon usage indices averaged over the whole gene sequence. To explore this possibility, we examined the correlation between resistance and localized codon usage within the *aacC1* gene, in different ways.

First, we correlated our resistance levels with codon usage indices calculated over 30 bp sliding windows and identified significant positive correlations between resistance levels and COUSIN in *P. aeruginosa* and between resistance levels and |Δ(GC3)| in * A. baylyi* for some windows, mirroring the results at the whole gene scale. Additionally, some windows showed positive correlations between CAI and resistance level in *P. aeruginosa* and negative correlations between resistance and CAI and COUSIN in *A. baylyi* and between resistance and |Δ(GC3)| in *P. aeruginosa* (Fig. 4). These results validate the idea that there are localized codon usage effects that could not be identified using indices averaged over the whole sequence. The localized effects identified are not in the same direction and for the same index across species and do not concern the same parts of the protein. This suggests a species specificity in terms of sensitivity to local codon usage similarity. Additionally, there is no clear correspondence between sections of the gene that show local codon usage sensitivity and the secondary structure of the protein, making it difficult to generate further hypotheses about the mechanistic link (translation speed, translation fidelity and protein folding) between local codon usage and resistance level. A second way through which local codon usage could have an effect on translation is by the presence of rare codon bottlenecks. Consecutive rare codons have indeed been shown to increase translation errors and ribosome stalling and drop-off [79,80]. However, rare codon chains of different length and translation bottlenecks did not explain the resistance conferred by the *aacC1* synonymous variants in any of the three species used.

Finally, we explored the potential role of the presence of sequence motifs within the synonymous variants coding for information beyond the amino acid sequence, whose function and activity could interfere with transcription or translation of the synonymous variant (reviewed in [42]). In particular, the abundance of within-gene SD-like sequences had been shown to correlate negatively with protein production in *Methylobacterium extorquens* [77], and we hypothesized that a high frequency of anti-SD-like sequences in our variants could reduce translation efficacy and thus resistance. No effect of SD-like sequences was found in *E. coli* and *P. aeruginosa*, and contrary to expectations, we found a significant positive relationship between the abundance of anti-SD-like sequences and resistance in *A. baylyi*. This significance persisted even when the non-essential 5′ end of the CDS was removed, ruling out the potential positive effect of translation of a smaller, possibly more efficient protein. The biological determinant of this result is not clear and requires further examination. While the anti-SD sequence is commonly accepted to be conserved across bacterial species, similarly to the conservation of the 16S rRNA sequence [81], recent work (reviewed in [82]) puts into question the universality of the anti-SD sequence and the species-independent potential effect of within-gene anti-SD sequences by documenting within- and between-species anti-SD sequence diversity. We can thus not rule out that the anti-SD consensus sequence we used is not fully appropriate for *A. baylyi* or *P. aeruginosa*.

Our multi-species approach produced results suggesting that different species have different sensitivity to the various ways by which synonymous variation affects the phenotype. Whilst our results do not conclude that one codon index or sequence motif explains all of the phenotype diversity provided by synonymous variation, our findings do suggest that synonymous variation is acting on numerous levels simultaneously, in line with the diversity of synonymous mutation-related effects published in recent years. Indeed, variant sequence composition acts on translational initiation, translation elongation, mRNA decay, protein folding and protein error rate [30,34]. Additionally, as transcription and translation are coupled in bacteria [61], translation-related positive or negative feedback on transcription might add another layer of complexity.

 We reject the hypothesis that differences in synonymous variant phenotype are most explained by mRNA folding and its effects on translation initiation. Conservation of the first 30 bp of the CDS did not prevent large differences in resistance phenotype, and mRNA folding in the first 90 bp of the CDS did not explain significantly the resistance levels. We do not doubt the modulatory effect of this area and its impact on phenotype put forward in many recent papers [31,32, 44]. We do, however, note that it is only one of many synonymous sequence-related effects on phenotype.

In conclusion, our multi-species approach revealed that synonymous variation affects the immediate success of HGT and is likely to be a factor orienting transfers. But our experimental results indicate that the compatibility between a variant and a species is mediated through a diversity of mechanisms, some of which are not clearly understood yet, such that it is not possible for now to make a direct prediction of the resistance/activity level conferred from the nucleotide sequence of a gene. A full understanding of how the sequence characteristics determine the phenotype would allow for these sequence characteristics to be used as an input variable in general microbiota models [83], to predict resistance gene and plasmid circulation. This study also pointed at species-specific PCN as a factor determining resistance level, immediate HGT success and the orientation of gene transfers.

## Supplementary material

10.1099/mic.0.001652 Uncited Supplementary Material 1.

10.1099/mic.0.001652 Uncited Supplementary Material 2.

## Abbreviations

AUC: area under the curve
BH: Benjamini–Hochberg
BH: Benjamini–Hochberg
CAI: Codon Adaptation Index
COUSIN: COdon Usage Similarity INdex
CUB: codon usage bias
CUPs: codon usage preferences
CUTs: codon usage tables
FDR: false discovery rate
HGT: horizontal gene transfer
PCN: plasmid copy number
RBS: ribosome binding site
RE: regulatory elements
SD: Shine–Dalgarno
tAI: tRNA Adaptation Index
