# Supplementary material for: Hurdles to horizontal gene transfer: species-specific effects of synonymous variation and plasmid copy number determine antibiotic resistance phenotype
Source: Microbiology (Reading). 2026 Jan 16;172(1):001652. doi: 10.1099/mic.0.001652 (PMC12811044; doi:10.1099/mic.0.001652)
Supplement: Uncited Supplementary Material 1. [file mic-172-01652-s001.pdf]

Supplementary Materials

Supplementary Materials and Methods:

E. coli K12 MG1655 competent cells were mixed with 5ng of plasmid DNA for 15 minutes @4°C after which the samples were subject to 2000V for 5ms in an Eppendorf Eporator (VWR). Samples were recovered in 1 ml of LB media for 1hr @37°C, 220 rpm. Samples were then grown overnight on selective media (kanamycin 50 µg/mL). A single colony was isolated, grown overnight in 4 ml of LB and archived in glycerol at -80°C.

P. aeruginosa PAO1 (ATCC 15692) competent cells were mixed with 5ng plasmid DNA for 15 minutes @4°C after which the samples were subject to 2500V for 5ms in an Eppendorf Eporator (VWR). Samples were recovered in 1 ml of LB media for 1hr at 37°C, 220rpm. Samples were then grown overnight on selective media (gentamicin 50 µg/mL). A single colony was isolated, grown overnight in 4 ml of LB and archived in glycerol at -80°C.

A. baylyi ADP1 was cultured from frozen glycerol stocks overnight at 30°C, 220rpm. 1 ml of fresh culture was mixed with 100 ng of plasmid DNA and incubated overnight at 30°C, 220rpm, exploiting *A. baylyi'*s natural competency. The mix was then plated on selective plates (kanamycin 10µg/mL) and grown overnight at 30°C, 220rpm. A single colony was isolated, grown overnight in 4 ml of LB and archived in glycerol at -80°C.

Supplementary Figures

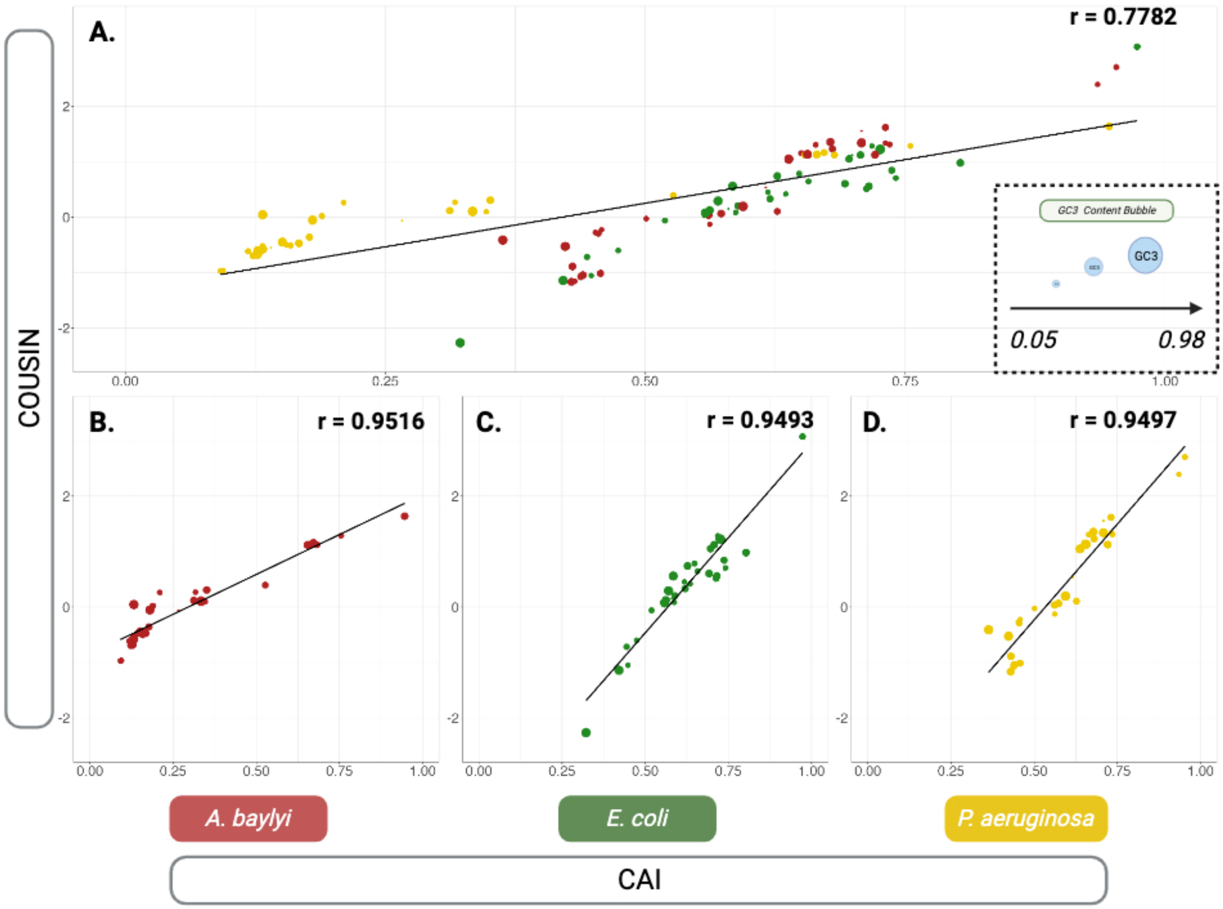

Figure S1: Correlation between CAI and COUSIN for the 32 *aacC1* synonymous variants. (A) Pearson correlation between CAI and COUSIN in all species. GC3 content is represented by bubble size. (B) Pearson correlation between CAI and COUSIN in all *A. baylyi*. (C) Pearson correlation between CAI and COUSIN in all *E. coli*. (D) Pearson correlation between CAI and COUSIN in all *P. aeruginosa*.

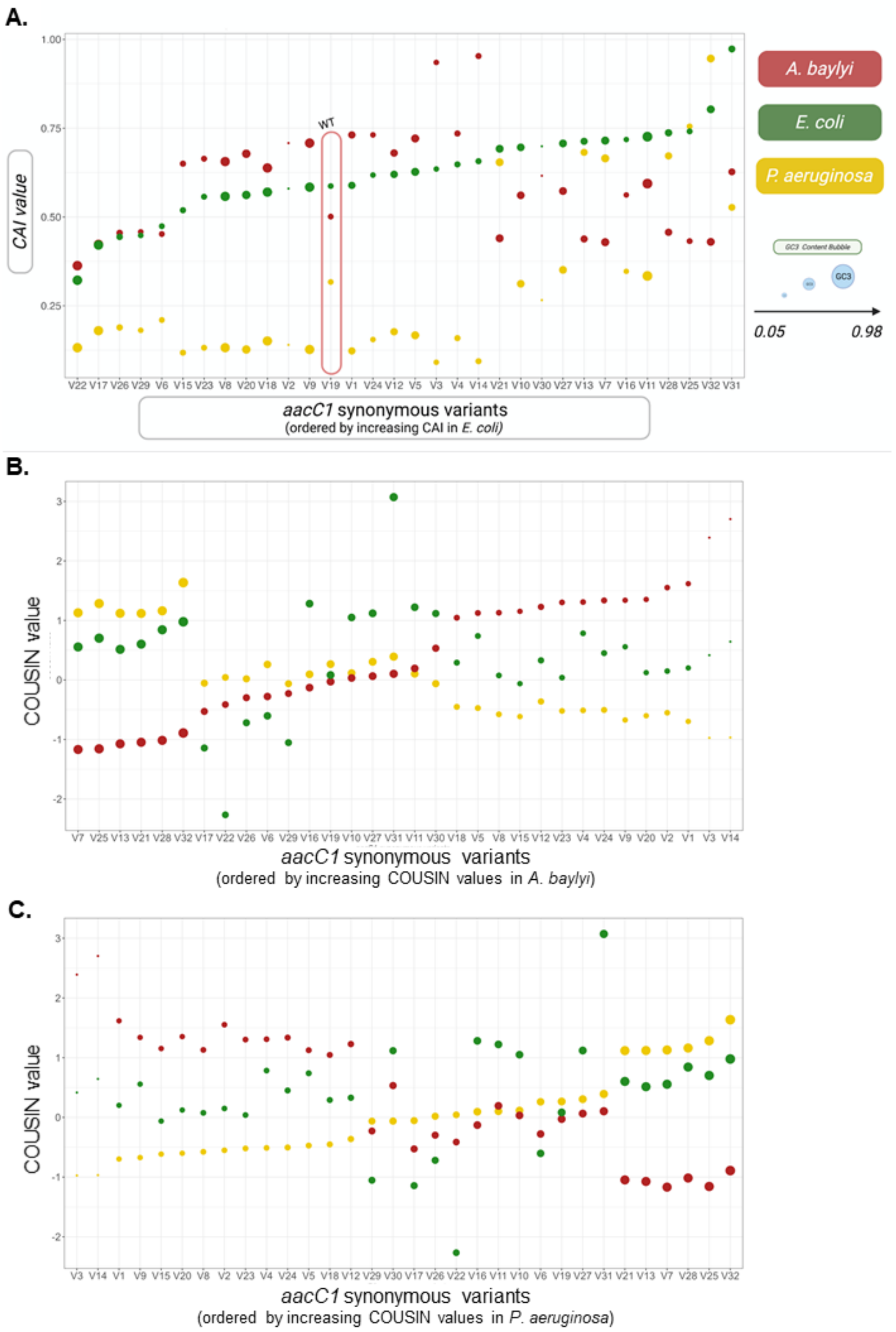

Figure S2: A. Codon Adaptation Index (CAI) for the 32 synonymous *aacC1* variants. Variants are ordered by increasing CAI values in *E. coli*. **B.** COUSIN index for the 32 synonymous *aacC1* variants, ordered by increasing COUSIN values in *A. baylyi.* **C.** COUSIN index for the 32 synonymous *aacC1* variants, ordered by increasing COUSIN values in *P. aeruginosa.* GC content at the third base of each codon (GC3) is represented by bubble size in the three panels.

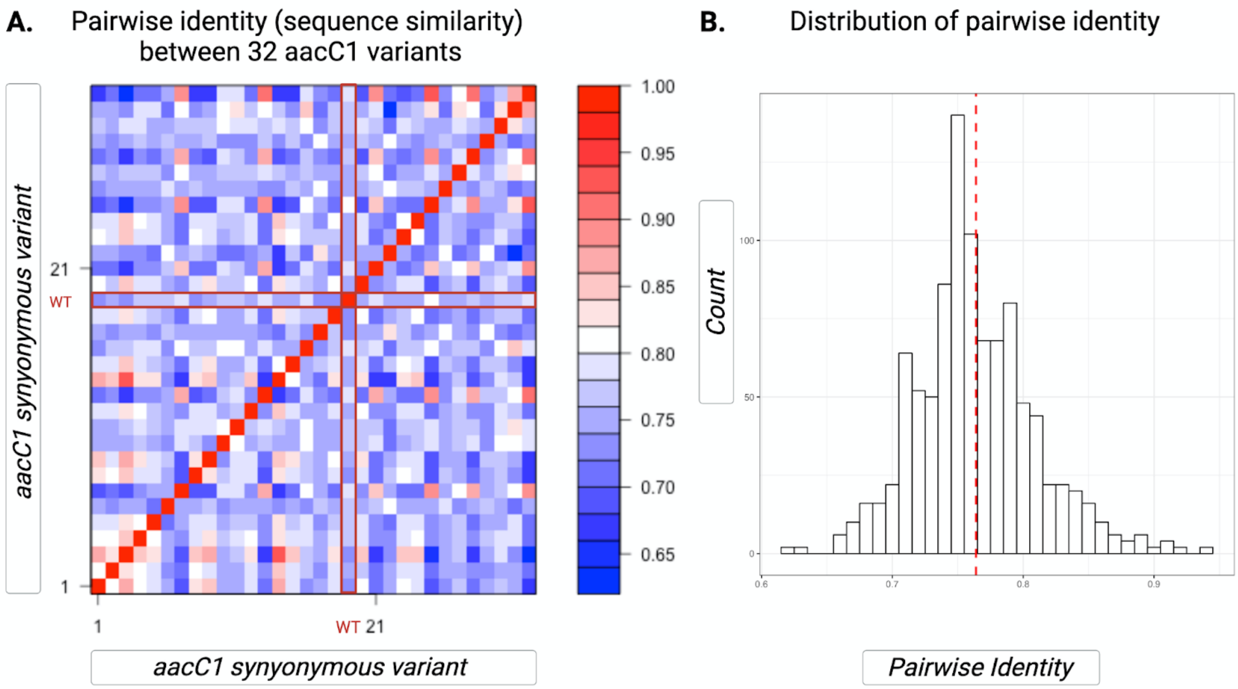

Figure S3: Pairwise identity of the 32 *aacC1* synonymous variants: (A) Heatmap of *aacC1* variants pairwise identity. Pairwise distance between the wt *aacC1* sequence, and the 31 synonymous variants is boxed in red. (B) Distribution of pairwise identities between the 32 synonymous variants. The red dotted vertical line represents the median pairwise distance.

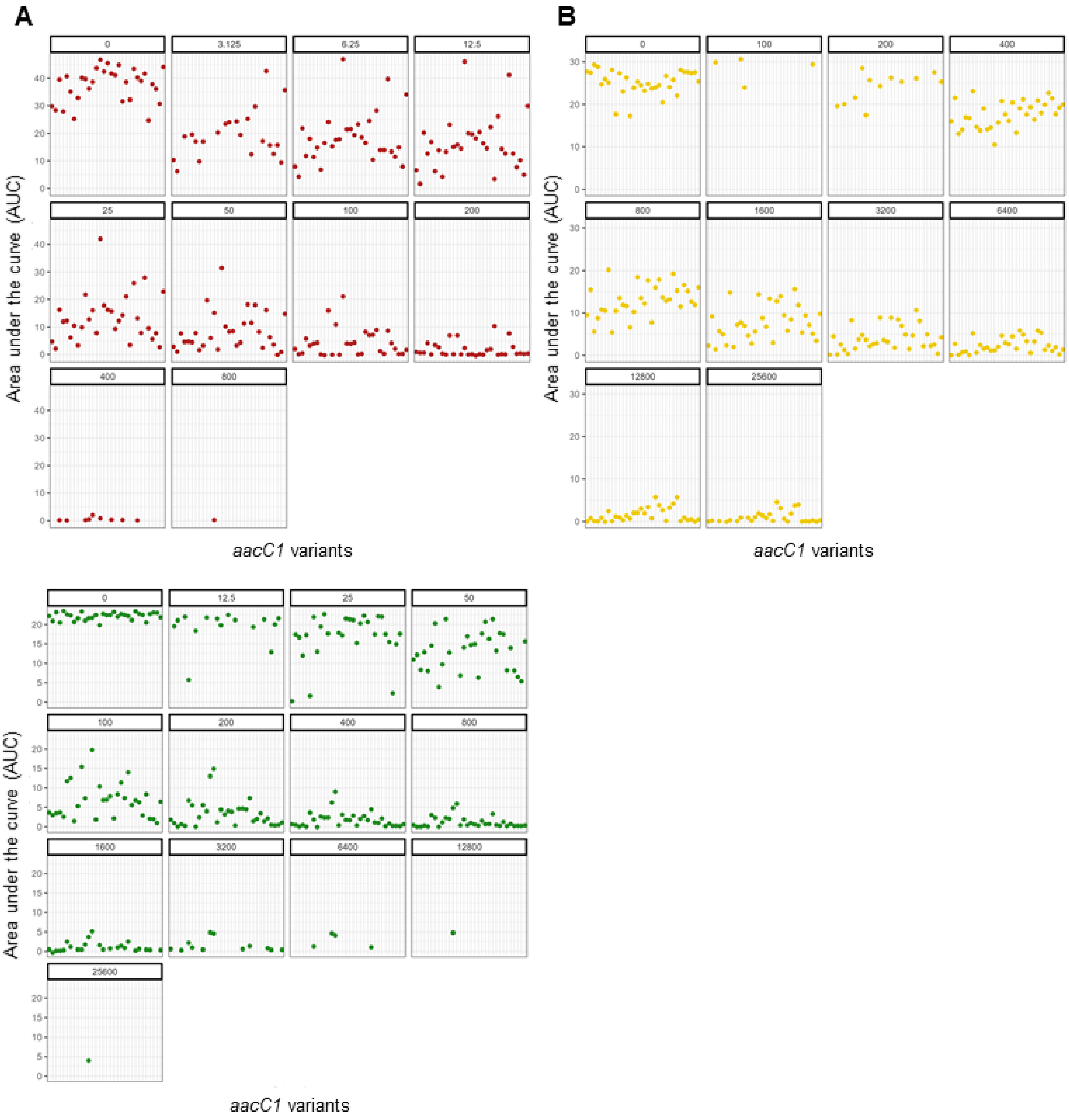

Figure S4. Area Under the Curve (AUC): Mean AUC values for each variant in each gentamicin concentration. Variants are ordered from 1-32 on the x avis. The wt variant is V19. **A.** *A. baylyi.* The graphs do not contain V3, V4, V12, V14, V20 or V32 as they could not be transformed into *A. baylyi*. **B.** *P. aeruginosa* **C.** *E. coli.* AUC of individual samples is available in supplementary file S13-15.

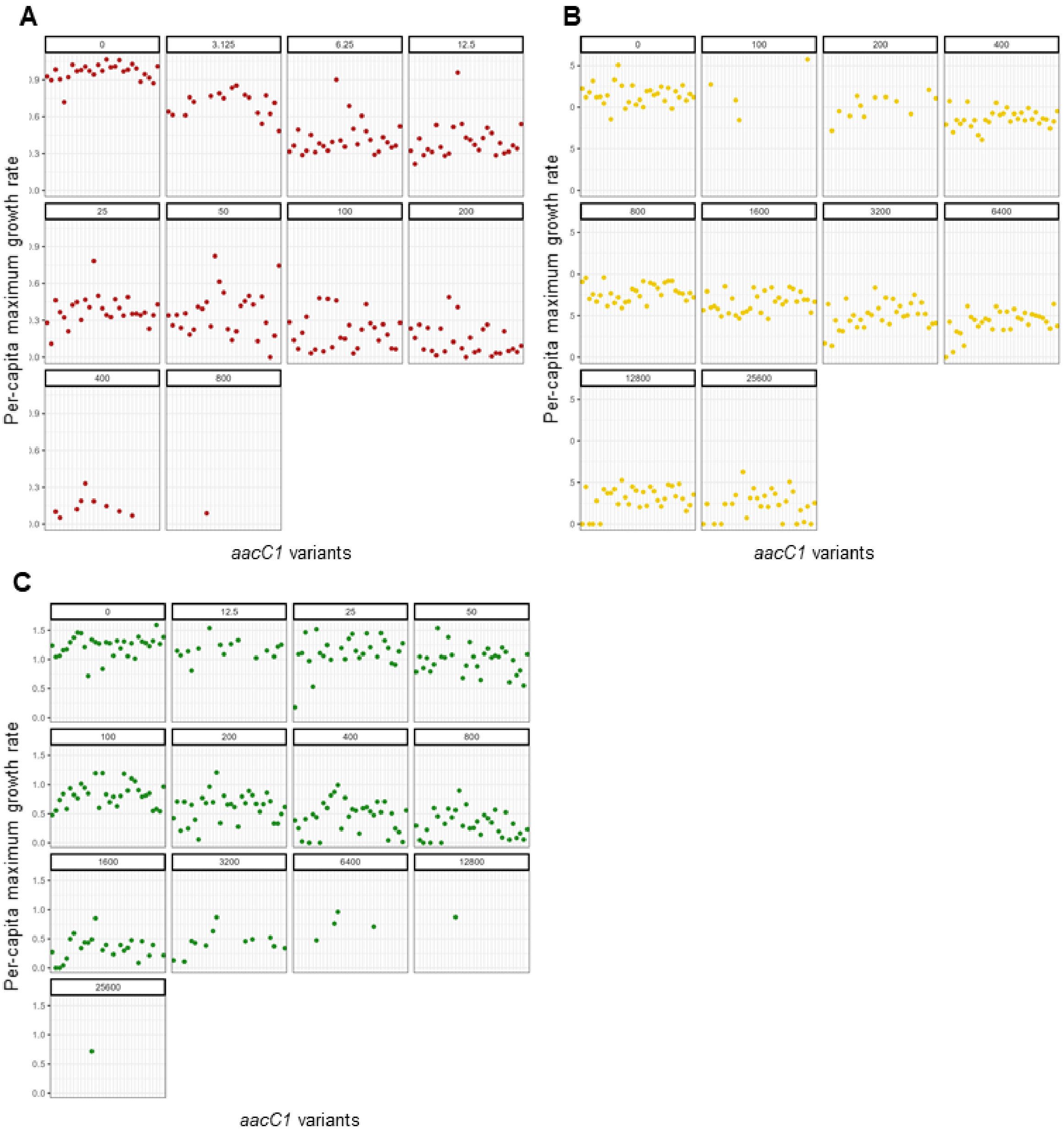

Figure S5. Per-capita maximum growth rate. Per-capita maximum growth rate for each variant in each gentamicin concentration. Variants are ordered from 1-32 on the x avis. The wt variant is V19. **A.** *A. baylyi.* Note that the graphs do not contain V3, V4, V12, V14, V20 or V32. **B.** *P. aeruginosa* **C.** *E. coli.* Max OD 600 individual sample data is available in supplementary file S13-15.

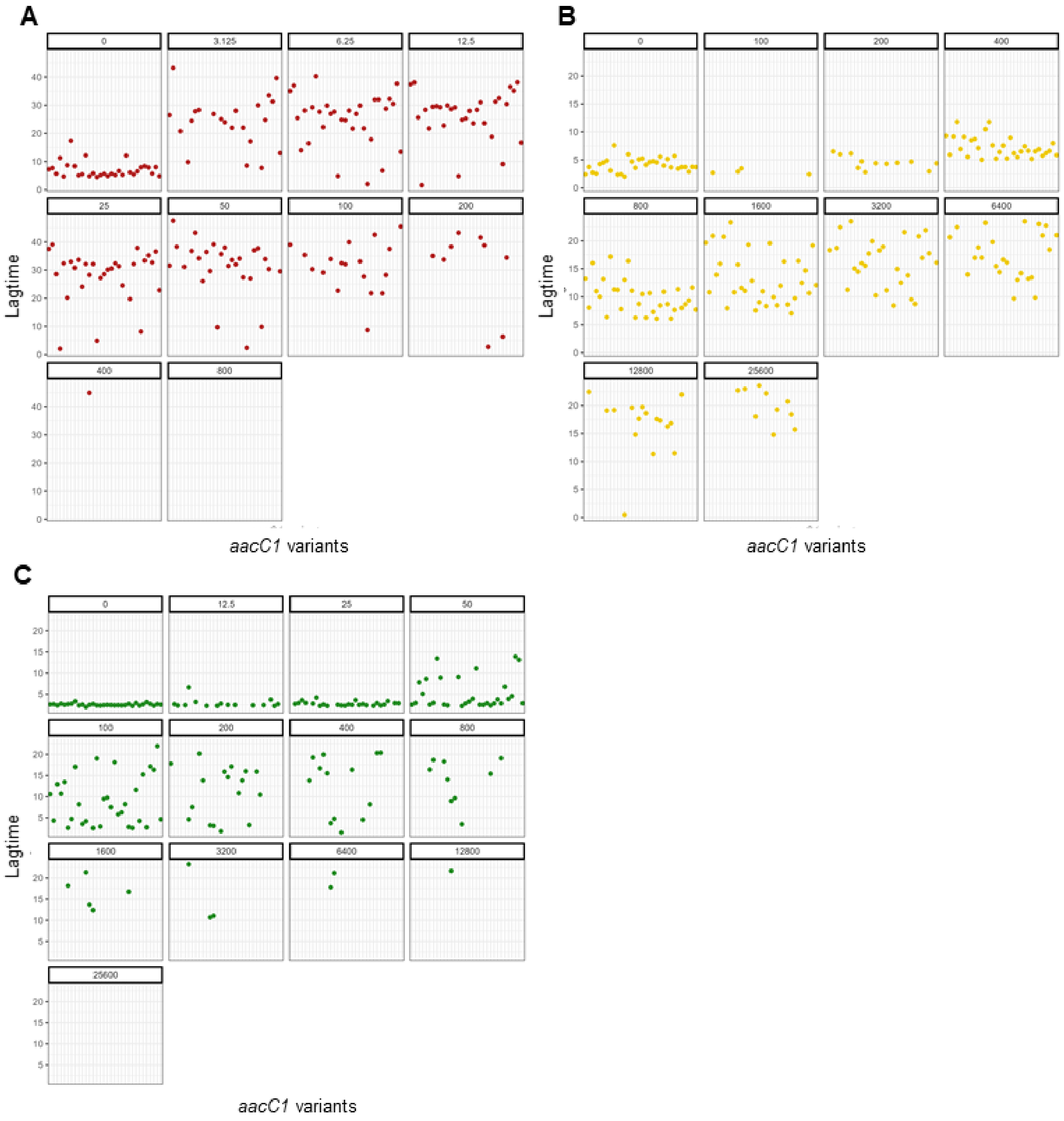

Figure S6. Lag Time Lag time calculated as the time needed for the bacterial culture to reach an OD 600 of 0.3. Variants are ordered from 1-32 on the x avis. The wt variant is V19. **A.** *A. baylyi*. Note that the graphs do not contain V3, V4, V12, V14, V20 or V32. *A. baylyi* cultures were grown for 48hrs. **B.** *P.aeruginosa* **C.** *E. coli*. Max OD 600 individual sample data is available in supplementary file S13-15.

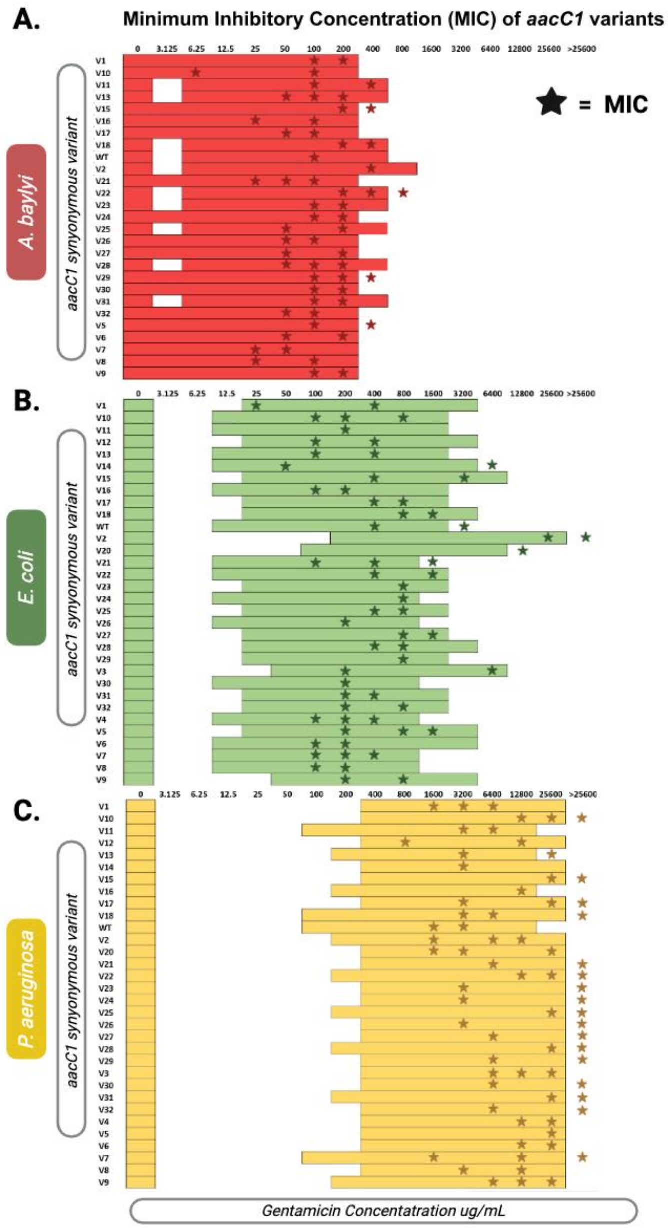

Figure S7: Minimum Inhibitory Concentrations of *aacC1* variants: Two-fold increases in gentamicin concentration are displayed on the x axis. aacC1 variants are represented on the y axis. Gentamicin concentration in which growth curves were performed are colored according to species. Non-measured concentrations are left blank. The MIC for each growth curve replicate is represented by a coloured star. MIC is defined here as the first concentration at which there is no visible growth (<0.1 OD 600). MIC stars outside of the coloured boxes represent samples that grew (>0.1 OD 600) in the respective highest measured gentamicin concentration. The next highest concentration is taken as the MIC and should be read as >Highest Measured Concentration (ex. *A. baylyi* V15 MIC = >200µg/mL gentamicin). ***A.*** *A. baylyi.* **B.** *E. coli.* **C .** *P. aeruginosa.*
